# Operation for Abscess of the Spleen

**Published:** 1888-04

**Authors:** 


### OPERATION FOR ABSCESS OF THE SPLEEN.

Dr. Carl Lauenstein, of Hamburg, reports a very interesting case in the *Deutsche medicinische Wochenschrift*, of December 22, 1887, the first case, in fact, of a cutting operation for abscess of the spleen on record.

The patient was a coppersmith, 23 years of age, who entered the Seaman's Hospital, in Hamburg, on September 17, under a diagnosis of typhoid fever, after having been sick for fourteen days. He was in a febrile condition, with a pulse of 96, tongue red at the edges and white in the centre, had been suffering with diarrhœa, and his abdomen was somewhat tympanitic. Splenic dullness was very much increased, extending in the axillary line from the lower border of the fifth rib forwards and downwards to the free border of the ribs, and backwards to within less than an inch of the spinal column. From the middle line forwards it encroached on the region of normal cardiac dullness, and over the somewhat enlarged dull region of the left lobe of the liver. The spleen was not palpable through the abdominal wall, since the patient did not use the diaphragm in respiration, and during inspiration there was some retraction of the epigastric region instead of expansion of the abdomen. The patient complained somewhat of pain in the splenic region, though there was no sensitiveness on pressure. There was no pulmonary abnormality, except an unusually high position of the diaphragm, and some friction sounds at the boundary of the base of the left lung.

Under a fluid diet, wine and hydrochloric acid internally, and the wet pack externally, the condition of the patient was not materially changed. The temperature fell in the morning, and rose in the evening to 39° C. Three days after entering the hospital the patient had a severe rigor, with a rise of temperature to 40° C., very small pulse, 128, and cyanotic lips. He then complained of increased pain in the splenic region, the dullness of which was not increased forwards and downwards, but above and behind was markedly increased. The dullness reached about one and a half inches above the angle of the scapula, and had an extent of thirty-five by eighteen cm. With the exception of the fixed condition of the diaphragm, and the marked friction sound at the base of the left lung, there was no abnormal thoracic condition.

On the day after the rigor there was a severe collapse, with a fall of temperature to 35.4°C., small pulse of 72, and coldness of the extremities, from which the patient was aroused only after the use of stimulants and heat. As the pain in the splenic region continued after the rigor, Lauenstein concluded that there was a purulent collection in the spleen; and on the fifth day after the patient entered the hospital he operated.

After the patient was anæsthetized the needle of the Dieulafoy aspirator was inserted in the eighth interspace in the axillary line, at the site of spontaneous pain, and about the middle of the anterior portion of the splenic dullness. The needle was pushed perpendicularly downward about two fingers' breadth, but no fluid was obtained until it was withdrawn carefully about two cm., when a small quantity of chocolate-colored fluid escaped, which had a penetrating fœtid odor. The needle was kept in place, and in order to empty the abscess thoroughly about ten cm. of the ninth rib were resected, this rib being selected in order to avoid wounding the pleura. In order to avoid hæmorrhage the thermo-cautery was used to cut down under the resected rib and parallel to it. Though the spleen was lying close to the diaphragm it was not adherent to it at the place of incision. After the cautery was carried down about two cm. into the splenic tissue a chocolate-colored fluid welled up by the side of the cautery. The incision in the spleen was now widened to the extent of the resection of the rib, and the borders of this incision were brought up against the wall of the thorax and fixed there by means of sharp hooks.

When the finger was introduced into the incision in the spleen it entered a cavity about the size of a goose's egg, the walls of which were very friable, and in which several loose fragments of tissue were found, that were removed with forceps. The cavity was washed out with cold salicylic acid solution, and then tamponed with iodoform gauze; the whole was then covered with an antiseptic dressing. Microscopic examination showed that the pus contained in the cavity was composed of pus-cells, some red corpuscles, fatty acid crystals, and crystals of hæmatin. No pus cocci or typhoid bacilli were found in the pus. The dressing was renewed on the fifth day after the operation, when it was seen that the spleen was adherent to the thoracic wall and to the diaphragm. Splenic dullness was at this time much lessened. For several days after the operation the temperature was about normal, but then rose, and the subsequent course of the illness was that of a case of typhoid fever. On October 11, the patient was entirely free of fever, but had the characteristic paleness, emaciation, and ravenous appetite of a typhoid convalescent. At the middle of October the cavity in the spleen had filled with granulations, and the thoracic wound was almost cicatrized. When the patient left the hospital on November 5, the area of splenic dulness was about 16 x 11 cm.

Lauenstein thinks it likely that the abscess was embolic, and it is known that embolic abscesses are most frequent in the course of the infectious diseases, such as scarlatina, diphtheria, typhoid fever, and pneumonia. Without the operation the termination of this case could scarcely have been otherwise than fatal, from rupture and purulent peritonitis, or rupture into the pleural cavity. Lauenstein thinks that in this case the abscess was one arising in the course of typhoid fever, and that the most rational and most surgical treatment was the one adopted. For the treatment of such abscesses conclusions must be drawn from the results of treatment of abscesses of other internal organs, especially of the liver. The best results in abscess of the liver have not been obtained by puncture or aspiration, but by free antiseptic incision, the mortality of the Lindemann-Landau and Volkmann operations, which are by free openings, being, according to Lauenstein, only 4.7 per centum. While Barbieri and Parzewski cured two cases of abscess of the spleen by puncture and aspiration, in both cases the splenic enlargement and abscess were accessible through the abdominal walls, which was not true in Lauenstein's case.

Lauenstein calls attention to the two important steps in his operation :

1. Puncture before incision, by which the danger of an escape of the purulent contents from the distended cavity was avoided.

2. Leaving the cannula in the abscess until the opening was completed, so that it served for a guide foı the thermo-cautery. He also regards it as a wise provision of nature that fixed the diaphragm so completely before the operation. Had the diaphragm continued to work as usual it is almost certain that the abscess would have been ruptured, and the contents extravasated. The circumscribed pleuritis, also, at the base of the left lung was a consideration, since it caused adhesion between the diaphragm and the costal pleura, without which the pleural cavity would probably have been opened at the operation, which would have seriously complicated matters.

Lauenstein gives the following points in the diagnosis of abscess of the spleen, besides the presence of an infectious disease, changes in temperature, rigors, etc. :

1. Enlargement of the area of dullness.
2. Spontaneous painful sensations.
3. Inflammatory phenomena in the region of the spleen, basal pleuritis, and fixed position of the diaphragm. Fluctuation must not be waited for, since it can be detected only in case the enlargement is below the free border of the ribs.

